# Hypnotism

**Published:** 1891-01-03

**Authors:** 


### HYPNOTISM.

Hypnotism has greatly exercised the minds of both medical men and the public during the past year. It is flattering to our British strength of mind that fewer people in this country are found to be fit subjects for hypnotism than is the case in France; and it is a tribute both to the intelligence and the conscientiousness of British medical men that they regard the whole subject with wholesome suspicion. In France both doctors and patients seem disposed to hold hypnotic treatment as of great importance. In our judgment if hypnotism were never heard of more the world would not be the loser. Even in France medical men are by no means agreed as to the use to be made of the treatment. Dr. Liebeault, of Nancy, seems to have given himself up to hypnotising as if it were the only resource of the medical art. The distinguished Charcot, on the other hand, considers that only hysterical persons can or ought to be hypnotised. The indiscriminate use of such a peculiar method of treatment he warmly condemns. Dr. Lloyd Tuckey, in this country, we believe, still practises hypnotically, and probably a limited number of other persons. The present state of the British medical judgment is perhaps this: that the phenomena of hypnotism are undoubtedly real and not to be denied; that certain hysterical persons of both sexes may be treated hypnotically with present advantage, and possibly without future injury; but that hypnotism is no more to be practised, except in cases of extreme necessity, than is chloralism, morphinism, or any other method of treatment of which the disadvantages are almost as great as the benefits. We congratulate ourselves and our fellow-countrymen that our nerves are as yet so firm and steady that hypnotism is but a curiosity amongst us. Long may it remain so!

